# Spatial distribution of *Glossina morsitans* (Diptera: Glossinidae) in Zambia: A vehicle-mounted sticky trap survey and Maxent species distribution model

**DOI:** 10.1371/journal.pntd.0011512

**Published:** 2023-07-27

**Authors:** Jackson Muyobela, Christian W. W. Pirk, Abdullahi A. Yusuf, Catherine L. Sole

**Affiliations:** 1 Department of Zoology and Entomology, University of Pretoria, Hatfield, Pretoria, South Africa; 2 Department of Veterinary Services, Tsetse and Trypanosomiasis Control Unit, Ministry of Fisheries and Livestock, Lusaka, Zambia; University of Cincinnati, UNITED STATES

## Abstract

**Background:**

Tsetse-transmitted African trypanosomiasis is a debilitating and fatal disease of humans and livestock if left untreated. While knowledge of the spatial distribution patterns of tsetse is essential for the development of risk-based vector control strategies, existing distribution maps in Zambia are more than 40 years old and were based on coarse spatial resolution data. The recently developed vehicle-mounted sticky trap (VST) provides an alternative sampling device to aid in updating existing distribution maps but has not been applied outside an experimental setting and is limited to motorable tracks. Therefore, the objective of the present study was to demonstrate the effectiveness of utilizing the VST for area-wide surveys of *Glossina morsitans* and to use the occurrence records to predict its spatial distribution in Zambia under current environmental conditions using Maxent.

**Methodology/Principal findings:**

Two-sided all-blue VST baited with butanone and 1-octen-3-ol was used to survey 692 and 1020 km of transect routes in *G*. *m*. *centralis* Machado and *G*. *m*. *morsitans* Westwood previously published distribution in Zambia. Maxent species distribution technique was used to predict the potential distribution of the two subspecies using current climatic and environmental data which was then compared to the historical distribution. A total of 15,602 tsetse were captured with *G*. *m*. *morsitans* (58%) being the most abundant. *G*. *m*. *centralis* and *G*. *pallidipes* Austin represented 39 and 2% of the catch respectively, and *G*. *brevipalpis* Newstead was also detected. The predicted potential distribution for *G*. *m*. *centralis* was 80,863 km^2^ while that of *G*. *m*. *morsitans* was 70,490 km^2^ representing a 47 and 29% reduction compared to their historical distributions, respectively.

**Conclusion/Significance:**

The VST is effective for sampling *G*. *morsitans* outside experimental settings and is recommended for use as an additional tsetse survey tool. The spatial distribution of *G*. *morsitans* in Zambia has reduced by 101,051 km^2^ due to temperature and land cover changes.

## Author summary

Tsetse habitat is increasingly fragmented owing to anthropogenic pressures and climate shifts which leads to spatial variability of African trypanosomiasis burden. Mapping tsetse occurrence is therefore a useful predictor of disease risk and provides the basis of evidence-based vector control strategies. In this study, we demonstrate the suitability of using the recently developed vehicle-mounted sticky trap for the rapid generation of occurrence records that can be used in ecological niche models to predict the distribution of *G*. *morsitans*. Since resources for vector surveillance and control are generally scarce in endemic settings, this alternative survey approach may be more efficient in the identification of priority areas for vector intervention.

## Introduction

Tsetse (Diptera: Glossinidae) occur in sub-Saharan Africa and are the only biological vectors of trypanosomes that cause human African trypanosomiasis (HAT or sleeping sickness) and animal African trypanosomiasis (AAT or nagana). Trypanosomiasis remains a debilitating and fatal disease if left untreated. In livestock, AAT causes annual losses of over 4 billion USD due to increased cattle and calf mortality, reduced calving rates, and reduction in milk and meat sales [1]. Diagnosis of HAT is difficult and treatments are often challenging to administer [2]. Accurate knowledge of local tsetse spatial distribution patterns is essential to understanding trypanosomiasis epidemiology and transmission dynamics [3] and is vital to the development of risk-based vector control strategies [4].

The distribution of tsetse is influenced by climate [5], vegetation [6], and host availability [7]. Climate, particularly temperature, is an important driver of tsetse population dynamics affecting key aspects of tsetse ecology such as vector survival, pupal development, fecundity, and density [8]. Global temperatures were reportedly 1.31°C higher in 2017 from the last century [9], and hence, there is a need to understand how this change has affected spatial distributions of tsetse. Vegetation is important for maintaining microclimatic environments that provide suitable conditions for adult resting sites and puparial development [10]. In recent years, habitat fragmentation, defined as the breakup of native vegetation into smaller isolated fragments [11], has led to a notable modification of tsetse habitat as a result of anthropogenic activities [6]. Progressive clearing of natural vegetation for cultivation, the introduction of domestic animals, and the almost complete disappearance of large game animals have resulted in changes in tsetse distribution [12], particularly among savannah group flies [13]. These changes have important implications for disease epidemiology and need to be considered when developing area-specific evidence-based vector management strategies. Thus, regularly updating the distribution tsetse is essential.

**Fig 1:**
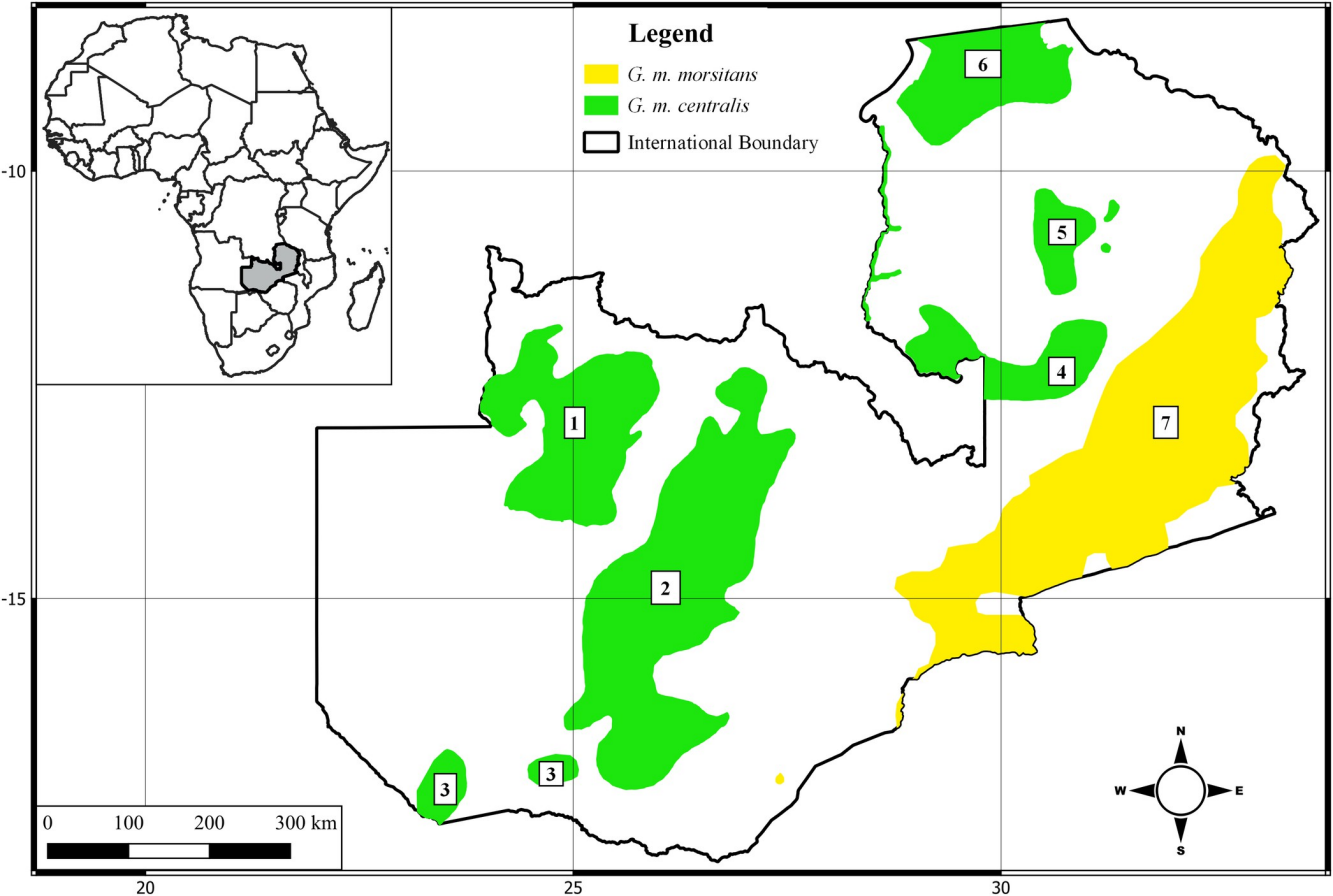
Historical distribution of the allopatric subspecies *G*. *m*. *morsitans* and *G*. *m*. *centralis* in seven distinct tsetse belts. Data on each subspecies in Evison and Kathuria [14]. Tsetse belt 3 was eradicated using sequential aerial spray technique in two separate operations in 2009 and 2014. The base map layer was obtained from the Database of Global Administrative Area GADM (https://geodata.ucdavis.edu/gadm/gadm4.1/shp/gadm41_ZMB_shp.zip)and under the license https://gadm.org/license.html. The figure was created using QGISv3.0 (http://qgis.org/en/site/).

Existing tsetse distribution maps in Zambia are more than 40 years old and were based on coarse spatial resolution data obtained from surveys done in the colonial era [14] and published by Ford and Katondo [15]. According to these maps, four species of *Glossina* occur in Zambia namely, *Glossina fuscipes martini* Zumpt, *G*. *brevipalpis* Newstead, *G*. *pallidipes* Austin, and *G*. *morsitans*. *Glossina morsitans* has the widest distribution covering an estimated 253,000 Km^2^ (or 38%) of the total land mass, with one subspecies, *G*. *m*. *morsitans* Westwood, occupying the hotter eastern part and the other subspecies, *G*. *m*. *centralis* Machado, occupying the cooler western and Northern part of the country [14] (Fig 1). The other three species exist within *G*. *morsitans* range but to a much lesser extent [14]. Being an efficient trypanosome vector, *G*. *morsitans* is therefore the most economically important tsetse species in Zambia, and most research and control efforts are directed against it. Recent information on the distribution of this tsetse species is however lacking.

A significant hindrance to surveying large areas infested with savannah tsetse has been the high cost of deploying baited stationary traps [16–18]. Area-wide tsetse surveys in large countries with a significant portion of their land mass infested with these tsetse are therefore not routinely undertaken [19]. The recently developed vehicle-mounted stick trap (VST) [20] provides an alternative effective sampling device that has been shown to rapidly detect the presence of *G*. *morsitans*. The VST could provide a suitable method of implementing low-cost area-wide surveys for regular updating of *G*. *morsitans* distribution. However, it is limited to surveying motorable tracks [20] and its utility outside an experimental setting has not been demonstrated.

One strategy to mitigate the accessibility limitation of VST tsetse surveys is to model its occurrence records using species distribution models (SDMs). Ecological niche models (ENMs) estimate the relationship between species occurrences and various environmental variables to produce prediction distribution maps [21–23]. Several statistical models have been used to predict species distributions [24,25], among which the maximum entropy model (Maxent) is effective for predicting species distribution locally and globally [26–28]. From a Bayesian perspective, the principle of maximum entropy states that subject to known constraints, the probability distribution that best represents the data is the one with the greatest entropy, thus the one which best reproduces the data [29]. Maxent aims to fit a penalized maximum likelihood model to presence-only species data that trades-off model fit and model complexity [29]. Maxent has been used to predict current [28] and future [30] distributions of tsetse.

The objective of the present study was to demonstrate the effectiveness of utilizing the VST for area-wide surveys of *G*. *morsitans* and to use the occurrence records to locally predict its distribution under current environmental conditions using Maxent. The predicted distribution will provide the basis for developing evidence-based tsetse and trypanosomiasis control strategies in Zambia.

## Materials and methods

### Ethical statement

Approval was granted by the Departments of National Parks and Wildlife (S3 Fig) and Veterinary Services Zambia to undertake entomological sampling in the game management areas and national parks.

### Study area

The study was conducted in Zambia, between the longitudes 22 and 34°E, and latitudes eight and 18°S. Much of the country consists of a plateau averaging from 1067 to 1220 m in elevation. Vegetation on the plateau is dominated by Miombo woodlands interspaced with riverside dambos (grassy wetlands) [31]. Mopane woodland is the dominant vegetation type in the valleys. A tropical climate prevails that is characterized by unimodal rainfall ranging from 500 to 1400 mm [32]. Rainfall is restricted to the period between November to April with temperature ranging from 14 to 30°C in the rainy season. May to July is cool and dry with temperature ranging from six to 26°C. August to October is hot and dry and temperature ranges from 17 to 35°C. Based on annual rainfall distribution, Zambia is divided into three agroecological zones namely Region I (less than 800 mm), Region II (800 to 1000 mm), and Region III (above 1000 mm) [33]. *G*. *morsitans* is known to occur in all three agroecological zones in eight distinct tsetse belts Fig 1 [14].

### Sampling device and materials

A two-sided all-blue (blue polyester, ParmaNet, Vestergaard Frandsen, Denmark) VST with one-sided adhesive film (Rentokil FE45, Liverpool, UK) was constructed according to the method described by Muyobela et al. [20]. Briefly, blue fabric was cut and fixed onto one side of two 5 mm × 1 m × 1.5 m plywood boards. To enumerate tsetse landing on trap panels, one-sided adhesive film was fastened above the cloth, using black duct tape. The trap was baited with butanone and 1-octen-3-ol which were dispensed at a rate of 150 mg/hr and 0.5 mg/hr respectively [34]. The trap was mounted on the back of a white Land Cruiser pick-up with its longest side horizontal to the ground. Non-stick baking paper was used to cover the sticky surface of the trap when not in use.

### Survey design and sampling

Stratified random sampling was used to determine the presence and absence of *G*. *morsitans* in the seven tsetse belts (Fig 1), except for tsetse belt 3, whose population was eradicated by sequential aerial spraying with ultra-low volume deltamethrin. Each sampled tsetse belt was stratified according to agroecological region to ensure that the entire environmental space occupied by the belt was sampled. Sampling routes were then located using QGIS version 3.0 and randomly selected. Selected routes were then uploaded to QField for QGIS android application which aided navigation in the field. The VST was operated by 3 individuals.

The VST traversed selected transects at a maximum speed of 20 km/h [20] making interval stops at 1 km to identify, sex, enumerate, and geo-reference captured flies. A one-minute waiting period was undertaken at a stop to allow the trapping of trailing tsetse before milking of the trap. VST traversed most transects from at least 5 km away from the historical limit of a tsetse belt and continued sampling for at least 20 km after detecting tsetse. Where tsetse was not detected, sampling continued for at least 60 km into the tsetse belt, and the route was sampled twice. The total distance surveyed was 692 and 1020 km for *G*. *m*. *centralis* and *G*. *m*. *morsitans* respectively. Sampling took place between September 2021 and August 2022. Actual sampling days consisted of 27 for *G*. *m*. *centralis* and 22 days for *G*. *m*. *morsitans*.

The results of a 1 km sampling interval were allocated to the mid coordinate of that km and were interpreted as a response from a 1 × 1 km area. Such an interpretation accounts for the daily displacement of *G*. *morsitans* which has been estimated to be between 167 m to 1.3 km [35] which plays a significant role in making the tsetse available for capture.

### Presence—Absence data

As described above, the VST survey involved continuous sampling along a transect. This sampling strategy is known to result in the loss of independence between samples located along the same transect [23], due to spatial autocorrelation. Geographic sampling bias can lead to environmental bias, resulting in an overrepresentation of environmental conditions associated with regions of higher sampling [36,37]. Constructing an ecological niche model with such data may fit the environmental bias, in addition to the niche signal, thus hindering model interpretation and application [38].

Moran’s I statistic was therefore used to assess the strength of spatial autocorrelation using the spdep package [39] in R [40]. As shown in Table 1, VST survey results for both *G*. *m*. *morsitans* and *G*. *m*. *centralis* were highly clustered (positive spatial autocorrelation). To reduce the effects of biased sampling while returning the signal of the species niche, spatial thinning of occurrence records for both subspecies based on nearest neighbour distance was done at 5 and 7 km using the spThin [41] package in R. Moran’s I test was reperformed on the thinned datasets, for both full (presence and absence) and p (presence only) data (Table 1). Based on observed Moran I Statistic, 5 km presence-only data were selected for modelling.

**Table 1:** Spatial autocorrelation analysis.

Subspecies	Data Type	No. of Records	Moran I Statistic	Expectation	Variance	Standard deviation	*P-value*
*G*. *m*. *morsitans*	VST raw full	1020	0.763	-0.0009	0.0002	50.699	< 2.2 e^-16^ *
	5 km thinned full	147	0.191	-0.0068	0.0013	5.505	1.85 e^-8^ *
	5 km thinned p	65	0.053	-0.0156	0.0028	1.288	0.10
	7 km thinned full	106	0.080	-0.0100	0.0017	2.149	0.02 *
	7 km thinned p	51	0.062	-0.0200	0.0033	0.454	0.32
*G*. *m*. *centralis*	VST raw full	692	0.567	-0.0014	0.0003	31.915	< 2.2 e^-16^ *
	5 km thinned full	106	0.182	-0.0095	0.0016	4.807	7.64 e^-7^ *
	5 km thinned p	35	0.027	-0.0294	0.0042	0.860	0.19
	7 km thinned full	79	0.057	-0.0182	0.0018	1.653	0.05
	7 km thinned p	23	0.047	-0.0454	0.0049	0.018	0.51

Full; full dataset with presence and absence records, P; dataset with presence-only records, *; statistically significant.

### Environmental variables and processing

Climatic and environmental predictors were used to estimate the species’ environmental relationship. Current climatic variables obtained were monthly minimum temperature, monthly maximum temperature, monthly average temperature, monthly precipitation, and 19 bioclimatic variables (bio1 –bio19) from WorldClim Global Climate Database version 2.1 [42]. Environmental variables included Moderate Resolution Imaging Spectroradiometer (MODIS) composite time series leaf area index (LAI) (MOD152H), normalised difference vegetation index (NDVI) (MOD13Q1), land surface temperature day (LST) (MOD11A1) [43], and land cover type 1 (LC) (MCD12Q1) [44] obtained from NASA’s EOSDIS Land Processes Distributed Active Archive Center [45]. The legend and class description of land cover type 1 (LC) (MCD12Q1) are given in Table 2. Data on human population density was obtained as Gridded Population of the World, Version 4 (GPWv4) from NASA’s Socioeconomic Data and Applications Center (SEDAC) [46]. Elevation data was obtained as Global 30 Arc-Second Elevation (GTOPO30) from the Earth Resources Observation and Science Center [47].

**Table 2:** International Geosphere-Biosphere Program (IGBP) legend and class descriptions of MODIS land cover type 1 (MCD12Q1).

Name	Value	Description
Evergreen Needleleaf Forests	1	Dominated by evergreen conifer trees (canopy > 2 m). Tree cover > 60%.
Evergreen Broadleaf Forests	2	Dominated by evergreen broadleaf and palmate trees (canopy > 2 m). Tree cover > 60%.
Deciduous Needleleaf Forests	3	Dominated by deciduous needleleaf (larch) trees (canopy > 2 m). Tree cover > 60%.
Deciduous Broadleaf Forests	4	Dominated by deciduous broadleaf trees (canopy > 2 m). Tree cover > 60%.
Mixed Forests	5	Dominated by neither deciduous nor evergreen (40–60% of each) tree type (canopy >2 m). Tree cover > 60%.
Closed Shrublands	6	Dominated by woody perennials (1–2 m height) > 60% cover.
Open Shrublands	7	Dominated by woody perennials (1–2 m height) 10–60% cover.
Woody Savannas	8	Tree cover, 30–60% (canopy > 2 m).
Savannas	9	Tree cover, 10–30% (canopy >2 m).
Grasslands	10	Dominated by herbaceous annuals (< 2 m).
Permanent Wetlands	11	Permanently inundated lands with 30–60% water cover and > 10% vegetated cover.
Croplands	12	At least 60% of the area is cultivated cropland.
Urban and Built-up Lands	13	At least 30% impervious surface area including building materials, asphalt, and vehicles.
Cropland/Natural Vegetation Mosaics	14	Mosaics of small-scale cultivation 40–60% with natural tree, shrub, or herbaceous vegetation.
Permanent Snow and Ice	15	At least 60% of the area is covered by snow and ice for at least 10 months of the year.
Barren	16	At least 60% of the area is non-vegetated barren (sand, rock, soil) areas with less than 10% vegetation.
Water Bodies	17	At least 60% of the area is covered by permanent water bodies.

For all monthly time series data, harmonic regression was performed using the TSA package [48] in R. Seven coefficients for each variable were extracted from the annual time series. The first coefficient in the regression is the mean of the variable and each further coefficient contributes to explaining the complete series by determining the amplitude and phase of the period that are half the length of the preceding period [49]. To generate uniform data structures for modelling, climatic and environmental predictors were resampled using the nearest neighbour method to give a spatial resolution of 1 km, reprojected to WGS84 coordinate reference system and spatially masked to the extent of Zambia, using the raster package [42] in R. All the environmental variables were converted into ASCII format.

### Model calibration

#### Pilot study

Environmental variable selection depends primarily on their restrictive effects on species distribution and spatial correlation [50]. In this study, variable classes, topographic, climatic, bioclimatic, and vegetation indices were run separately in Maxent software [51] using default settings. Variables contributing 10% and 4% or more to model gain were pre-selected for *G*. *m*. *morsitans* and *G*. *m*. *centralis* respectively. Selected predictor classes were then combined and rerun in Maxent with the same criteria used to select variables for each subspecies. Land cover type and human population density were included in both models as they are known to be correlated with tsetse habitat [10,12]. Pearson correlation analysis was then conducted on selected variables to assess the risk of multicollinearity among variables. For *G*. *m*. *morsitans*, all pairwise correlation coefficients were less than 0.7, and hence no risk of multicollinearity was observed [25] (S1 Fig). High correlation coefficient values (greater than 0.8) were however observed for some predictors of *G*. *m*. *centralis* (S2 Fig). Feng et al [52] showed that Maxent is robust to predictor collinearity in model training and that the strategy of excluding highly correlated variables has little impact because Maxent accounts for redundant variables. Therefore, all variables selected were included in the model due to their ability to improve model fit.

The ENMeval package [53] in R was used to test the sequential criterion of the lowest omission rate (OR) and the best area under the curve (AUC) to identify parameter settings providing the best model fit [54]. Feature combination and regularization multiplier were confirmed through this process.

#### Formal experiments

Occurrence data and selected environmental variables were uploaded into Maxent software. Various parameters were set, including ‘Create response curves’ and ‘Random seed’. Furthermore, 75% of the presence data was used to establish the model, and 25% was applied for testing, using bootstrap sampling, 20 replicates, 5000 maximum iterations, and 10 percentile training presence threshold rule (Table 3).

**Table 3:** Maxent parameter settings.

Option	Default Value	Setting Value
Randomly selected test set percentage	0	25
Regularization multiplier	1	0.5
Replicated run type	Crossvalidate	Bootstrap
Number of iterations repeated	1	20
Maximum number of repetitions Apply	500	5000
Apply threshold rules	None	10 percentile training presence
Features	Auto (hinge, product, quadratic, linear)	Auto (hinge, product, quadratic, linear)

The default feature setting of Maxent was chosen with a 0.5 regularization multiplier. Jackknife tests we used to measure variable contribution rates and importance. The log-log (cloglog) probability of presence maps produced were reclassified using the raster package in R and divided into four levels based on Jenks [30]: unsuitable area (0–0.097), marginally suitable area (0.097–0.323), moderately suitable area (0.323–0.603), and highly suitable area (0.603–1).

### Model evaluation

**Fig 2:**
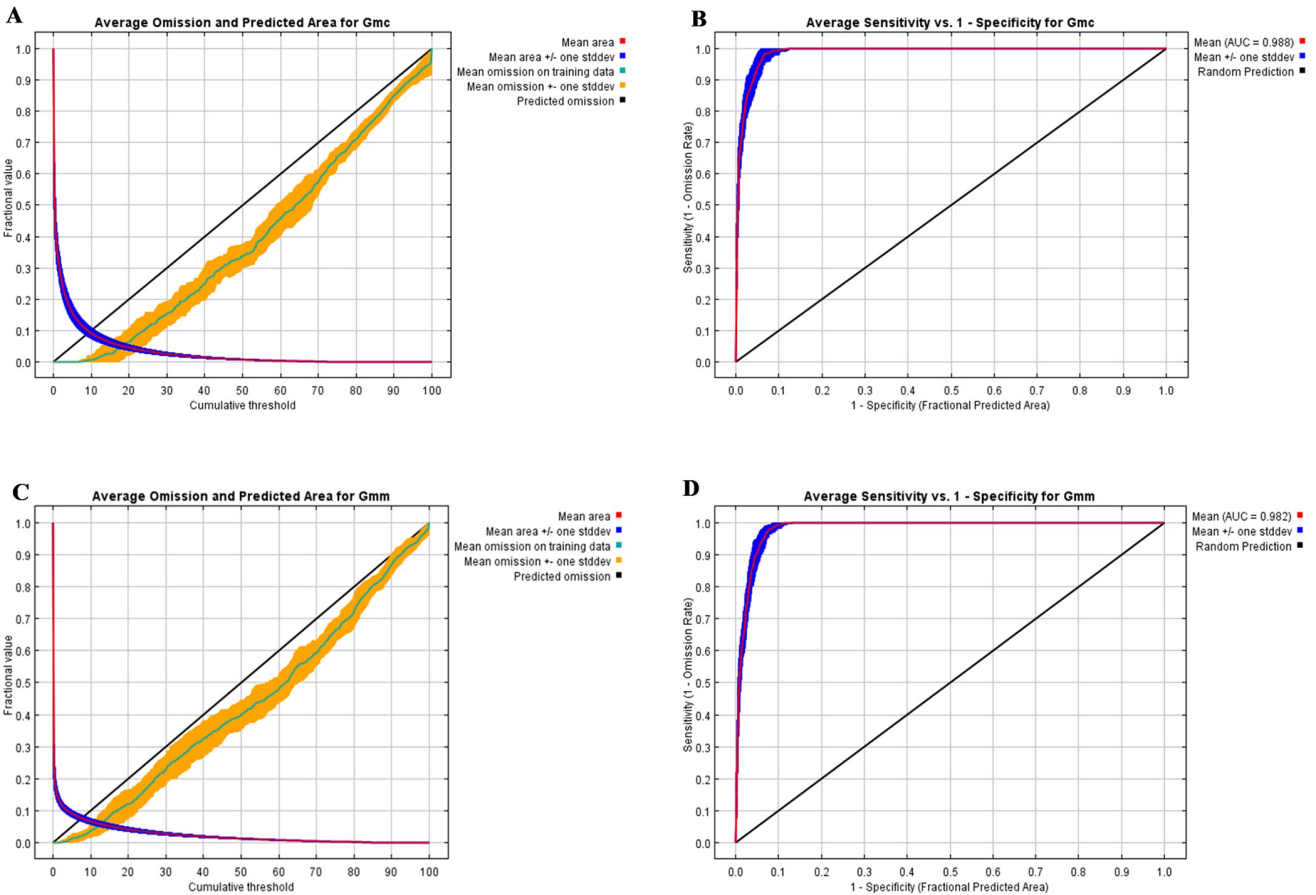
Validation charts of model performance. (A) *G*. *m*. *centralis* omission rate; (B) *G*. *m*. *centralis* operating characteristic curve; (C) *G*. *m*. *morsitans* omission rate; (D) *G*. *m*. *morsitans* operating characteristic curve.

The training omission rate for both subspecies was close to the predicted omission rate which indicated that the models were well-established (Fig 2A and 2C). The receiver operating characteristic curve (ROC) obtained by plotting Sensitivity (true presence rate) on the y-axis against 1- Specificity (false presence rate) on the x-axis plotted across all available thresholds was used to evaluate the models [26]. The area under the curve (AUC) of the ROC, whose value ranges from 0.5 to 1, provides a measure of model accuracy [55]. A value of 0.5 indicates that a model performs no better than random. AUC values of 0.5–0.7 represent poor model performance, values of 0.7–0.9 are considered moderate, and values above 0.9 indicate excellent model performance [26]. The mean AUC values of ROC were observed to be 0.988 ± 0.002 and 0.982 ± 0.002 for *G*. *m*. *centralis* and *G*. *m*. *morsitans*, respectively. This indicated excellent Maxent model performance for both subspecies (Fig 2B and 2D).

## Results

### Survey results

A total of 15,602 tsetse was captured during the survey (Table 4). The most abundant tsetse was *G*. *m*. *morsitans* (58%) followed by *G*. *m*. *centralis* (39%). *G*. *pallidipes* represented (2%) of the catch and was observed in *G*. *m*. *morsitans* range. *G*. *brevipalpis* was detected in *G*. *m*. *centralis* range in tsetse belt 6 (Fig 1). *G*. *m*. *morsitans* was detected in Tsetse belt 7. *G*. *m*. *centralis* was only observed in tsetse belts 2, 4, and 6 (Fig 1). No tsetse was detected in tsetse belts 1 and 5 despite 123 and 92 km of road being surveyed respectively. Historical limits of all tsetse belts were observed to have changed by generally receding towards the centre of the distribution.

**Table 4:** Tsetse catches characteristics.

Species	Male	Female	Total
*G*. *m*. *morsitans*	6,713	2,378	9,091
*G*. *m*. *centralis*	4,264	1,919	6,183
*G*. *pallidipes*	173	144	318
*G*. *brevipalpis*	11	0	11
**Total**	**11,161**	**4,441**	**15,602**

### Maxent model

#### Environmental variable contribution

The variables selected for modelling are shown in Tables 5 and 6 for *G*. *m*. *morsitans* and *G*. *m*. *centralis* respectively. As indicated, 7 variables were sufficient to model the former while 8 were required to adequately describe the potential distribution of the latter subspecies. For *G*. *m*. *morsitans*, isothermality, minimum temperature of the coldest month, and annual precipitation were observed to contribute the most to Maxent model gain (Table 5). For *G*. *m*. *centralis*, land cover type, human population density, and precipitation of the driest quarter, contributed the most to the model (Table 6).

**Table 5:** Predictor variables for *G*. *m*. *morsitans* and their contribution rates.

Abbreviation	Definition	Contribution Rate (%)
Bio3	Isothermality (%)	34.2
Bio6	Minimum Temperature of Coldest Month (°C)	22.2
Bio12	Annual Precipitation (mm)	11.5
LSTD6	Land Surface Temperature (°C) (Bi-Weekly Average)	11.2
Bio18	Precipitation of the Warmest Quarter	8.6
LC	Land Cover Type	8.0
H_popden	Human population density (number of persons per km^2^)	3.9

**Table 6:** Predictor variables for *G*. *m*. *centralis* and their contribution rates.

Abbreviation	Definition	Contribution Rate (%)
LC	Land Cover Type	31.1
H_popden	Human population density (number of persons per km^2^)	16.0
Bio17	Precipitation of the Driest Quarter (mm)	13.1
Bio3	Isothermality (%)	11.0
Bio15	Precipitation Seasonality (%)	9.3
LSTD1	Land Surface Temperature (°C) (Annual Average)	8.8
Bio8	Mean Temperature of the Wettest Quarter (°C)	6.1
Vapr	Water Vapour Pressure (kPa)	4.6

**Fig 3:**
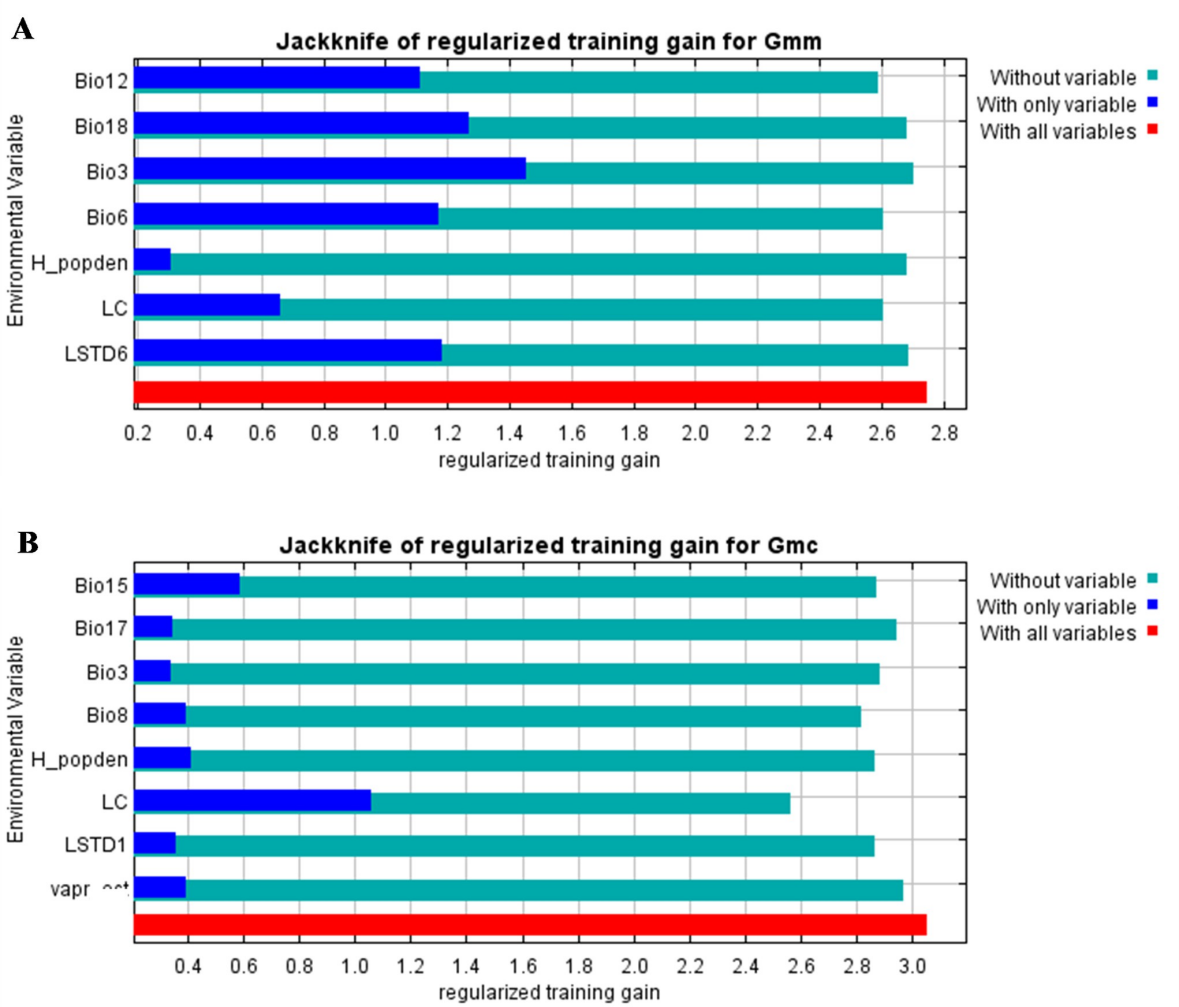
Jackknife tests for Maxent model variable importance. (A) *G*. *m*. *morsitans* model variable importance. (B) *G*. *m*. *centralis* model variable importance.

Jackknife test for variable importance revealed that for *G*. *m*. *morsitans*, the environmental variable with the highest gain when used in isolation was isothermality (Fig 3A). This indicated that isothermality had the most useful modelling information when used by itself. Annual precipitation was observed to decrease model gain the most when omitted (Fig 3A). This showed that it had the most information that wasn’t present in any of the other variables. For *G*. *m*. *centralis*, Jackknife test for variable importance revealed that land cover type had both the highest model gain when used in isolation and the highest decrease in gain when omitted (Fig 3B). Thus, it had the most useful information for modelling which was not present in any of the other variables.

**Fig 4:**
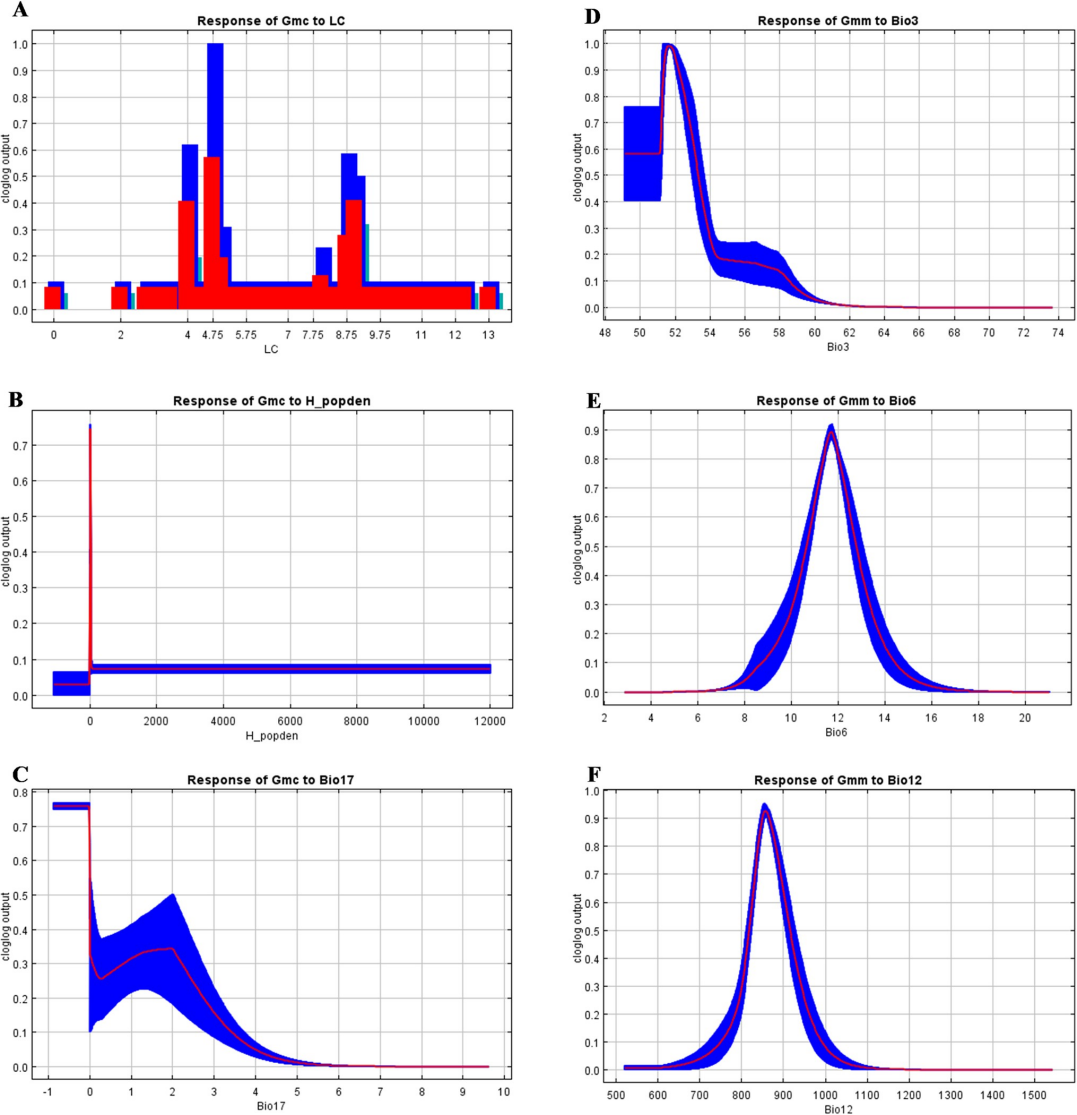
Response curves for dominant variables. (A) *G*. *m*. *centralis* response to land cover type (LC); (B) *G*. *m*. *centralis* response to precipitation seasonality (Bio15); (C) *G*. *m*. *centralis* response to precipitation of the driest quarter (Bio17); (D) *G*. *m*. *morsitans* to isothermality (Bio3); (E) *G*. *m*. *morsitans* response to minimum temperature of the coldest month (Bio6); (F) *G*. *m*. *morsitans* response to annual precipitation (Bio12).

Single-factor analysis was conducted on the three variables that contributed the most to Maxent model gain for both subspecies (Fig 4). According to Fig 4A and Table 2, *G*. *m*. *centralis* probabilities of occurrence were highest (100%) where more than 60% of the land cover was dominated by deciduous broadleaf trees having a canopy greater than two metres. Woody savannahs with tree cover ranging between 10 to 60% and a canopy of more than two metres were associated with moderate (60%) probabilities of occurrence for *G*. *m*. *centralis* (Fig 4A and Table 2). Increase in human population density from zero was associated with an abrupt decrease in *G*. *m*. *centralis* probabilities of occurrence (to less than 10%) (Fig 4B). A similar response was observed for *G*. *m*. *morsitans*. Low precipitation in the driest quarter (less than 3 mm) was observed to be associated with higher *G*. *m*. *centralis* probabilities of occurrence (Fig 4C). High probabilities of occurrence (100%) for *G*. *m*. *morsitans* were observed to be associated with moderate isothermality values (50–52%) with values greater than 60% reducing occurrence probabilities to 0% (Fig 4D). *Glossina m*. *morsitans* had a unimodal association with minimum temperature of the coldest month and annual precipitation (Fig 4E and 4F). The highest probabilities of occurrence were observed at 11.9°C and 850 mm for the minimum temperature of the coldest month (90%) and annual precipitation (92%), respectively. The highest probability of occurrence for *G*. *m*. *morsitans* was associated with woody savannahs with tree cover ranging between 10 to 60% having a canopy of more than two metres and being located near permanent water bodies.

#### Potential distribution

**Fig 5:**
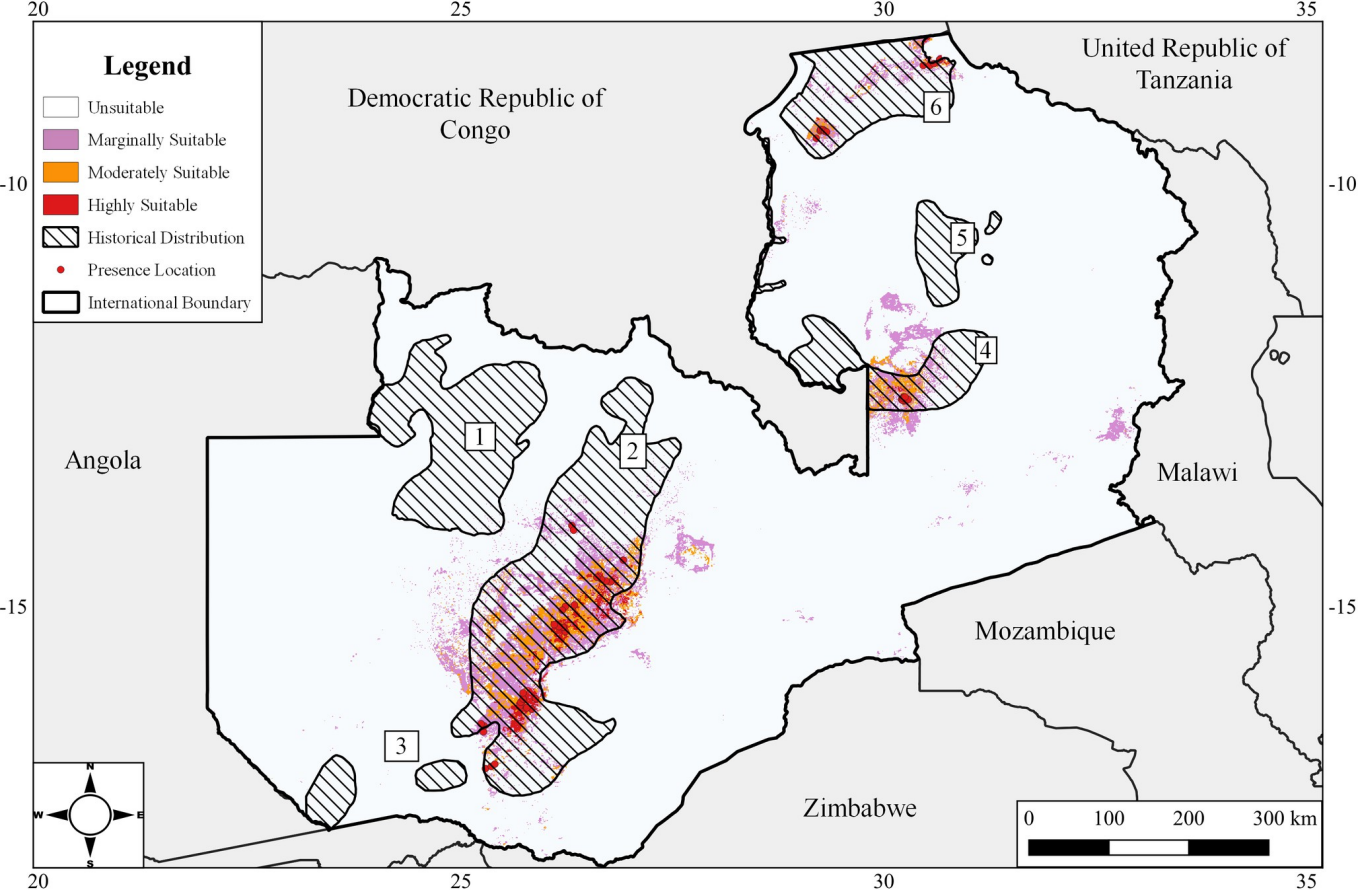
Historical distribution, presence locations, and potentially suitable area under current conditions of *G*. *m*. *centralis*. (Probability: unsuitable area 0–0.097; marginally suitable area 0.097–0.323; moderately suitable area 0.323–0.603; highly suitable area 0.603–1). The base map layer was obtained from the Database of Global Administrative Area GADM (https://geodata.ucdavis.edu/gadm/gadm4.1/shp/gadm41_ZMB_shp.zip) and under the license https://gadm.org/license.html. The figure was created using R.

**Fig 6:**
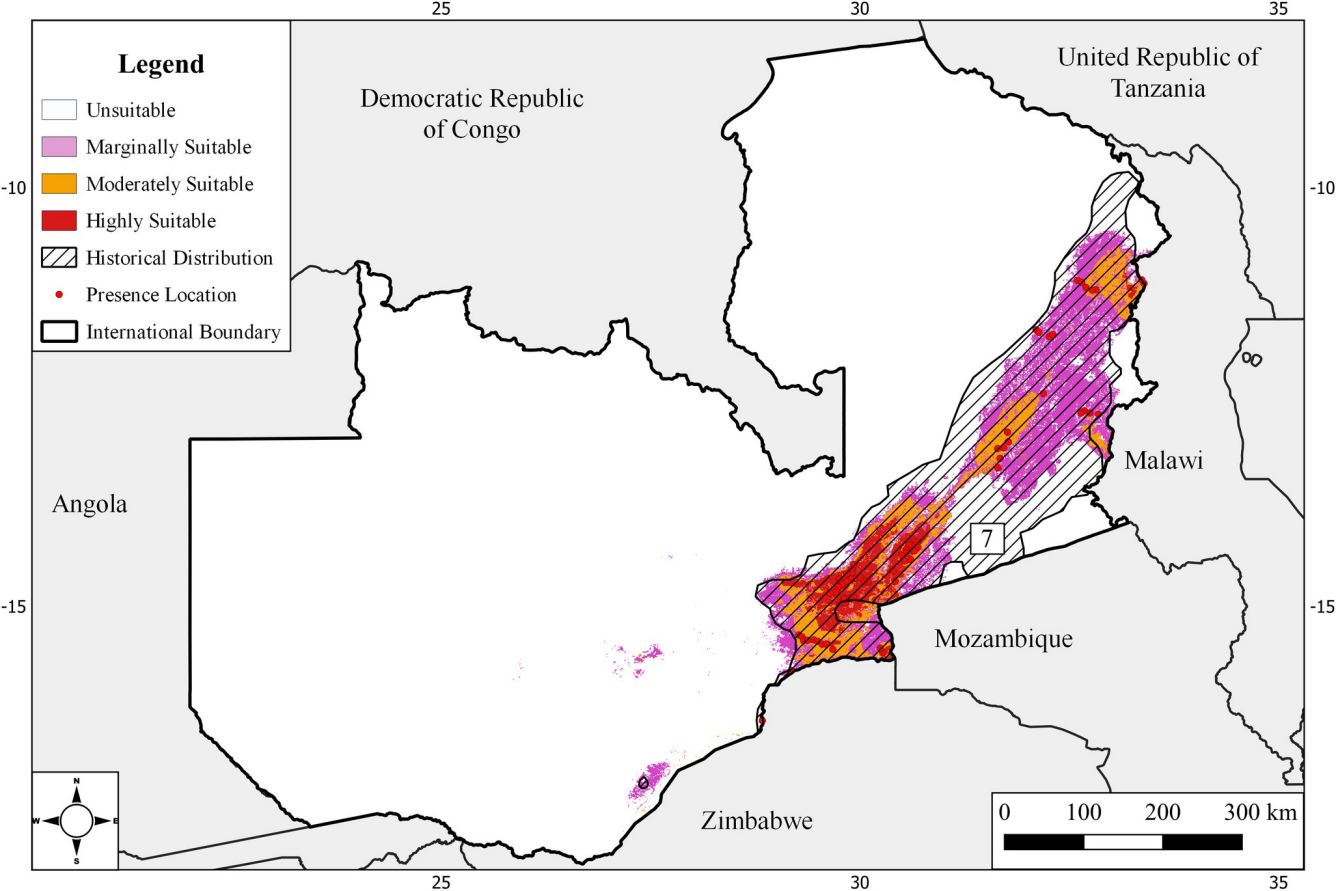
Historical distribution, presence locations, and potentially suitable area under current conditions of *G*. *m*. *morsitans*. (Probability: unsuitable area 0–0.097; marginally suitable area 0.097–0.323; moderately suitable area 0.323–0.603; highly suitable area 0.603–1). The base map layer was obtained from the Database of Global Administrative Area GADM (https://geodata.ucdavis.edu/gadm/gadm4.1/shp/gadm41_ZMB_shp.zip) and under the license https://gadm.org/license.html. The figure was created using R.

The predicted potentially suitable area for *G*. *m*. *centralis* and *G*. *m*. *morsitans* under current conditions are shown in Figs 5 and 6 respectively. About 80,863 km^2^ was identified as suitable habitat for *G*. *m*. *centralis* within and around its historical distribution. A small area of *G. m. centralis* habitat was observed in *G*. *m*. *morsitans* historical range. The predicted distribution represented an estimated 71,970 km^2^ (47%) reduction when compared to its historical distribution.

For *G*. *m*. *morsitans*, suitable conditions were predicted over 70,490 km^2^ within and around its historical distribution (Fig 6). The predicted suitable area represented an estimated 29,081 km^2^ (29%) reduction when compared to its historical distribution. The suitable area for *G*. *m*. *morsitans* was predicted as one tsetse belt with an isolated pocket in the southwest of the country.

## Discussion

Our results reaffirm the conclusions of Muyobela et al. [20] that the VST is an effective tool for the rapid detection and sampling of *G*. *morsitans*. As demonstrated here, VST is capable of sampling large areas in a relatively short space of time with minimal labour compared to other sampling tools currently available. These attributes render VST an essential tool for the rapid generation of critical information on *G*. *morsitans* distribution required to facilitate the planning of more detailed surveys or control operations [19]. Furthermore, our study showed that the tool could detect *G*. *pallidipes* and to a lesser extent *G*. *brevipalpis* despite being initially designed and optimised to sample *G*. *morsitans*. This result provides evidence that VST could be effective in sampling other savannah species of tsetse. We recommend studies to optimise VST design for sampling other economically important savannah tsetse.

An important limitation to the use of VST for tsetse surveys highlighted in this study was that species occurrence records obtained using this tool exhibit high positive spatial autocorrelation. Loss of independence among data causes parametric statistical testing procedures to give more significant results than what the data justifies, which is a serious problem for statistical and ecological interpretation [56]. We propose two approaches to ameliorate the effects of sampling bias of the VST based on the results of this study. Firstly, we recommend that sample transects should be set at a minimum distance of 5 km apart. This should nullify spatial autocorrelation between transects. Secondly, we recommend spatial thinning of occurrence records within a transect using the nearest neighbour distance as described by Aiello-Lammens et al. [41]. Random thinning at a 5 km radius was found to be sufficient to reduce spatial autocorrelation in our study, which is similar to the suggestion of Zhou et al. [30]. The implication for VST survey design is that transects should be long enough (at least 40 km) to retain sufficient sample locations for further analysis.

One of the primary objectives of conducting tsetse surveys is to determine the relative abundance or apparent density (AD) of species present in time and space [16]. Tsetse apparent density is defined as the number of flies caught per unit effort and is calculated as the number of flies caught per trap per day for stationary traps or the number of flies per section of fly round for fly round data [19]. Clearly, the computation of AD depends on the sampling tool used. For the VST, our study interpreted catches in a 1 km sampling interval as a response from a 1× 1 km grid. The implication is that catches in the 1 km interval should be reported as the number of flies per km^2^. To compute AD, we suggest accounting for the length of time the VST was operated in that km as it is part of the sampling effort. Thus, AD of the VST should be reported as the number of flies per km^2^ per unit time.

Our results agree with the general notion that temperature [30], precipitation [57], and land cover type [10], are important factors in determining the distribution of *G*. *morsitans*. Isothermality was found a significant contributor to the models describing the habitats of both *G*. *m*. *morsitans* and *G*. *m*. *centralis*, similar to the findings of Zhou et al [30]. Isothermality quantifies how large the day-to-night temperatures oscillate relative to the summer-to-winter (annual) oscillations [58]. An isothermal value of 100 indicates that the diurnal temperature range is equivalent to the annual temperature range, while anything less than 100 indicates a smaller level of temperature variability within an average month relative to the year. Being poikilotherms, both low and high temperatures affect tsetse survival, negatively influencing rates of mortality and fat metabolism in adults and pupae, and rates of larviposition and pupal development [59,60]. Our results suggest that *G*. *morsitans* prefers areas with stable temperatures similar to the findings of other researchers [5,59,60]. Stable temperatures (25–27°C) provide optimal conditions for pupal fat accumulation and development. Pupal fat reserves are exhausted before they complete development when exposed to a constant temperature below 16°C, while direct effects kill pupae before fat stores are exhausted at high temperatures (above 32°C) [59].

There is generally no record in the literature on the direct effects of rainfall on tsetse [57]. However, precipitation is thought to have two major indirect effects on tsetse survival; firstly, it maintains vegetation (tsetse habitat), and secondly, it causes local flooding which may drown pupae that are buried in loose soil causing depopulation [61]. Moderate annual precipitation was associated with high *G*. *m*. *morsitans* probabilities of occurrence as this level of precipitation favours the growth of Mopane woodland [62], the dominant vegetation type within this subspecies’ range. Deciduous Miombo woodland, which losses foliage in the hot dry season, is the dominant vegetation type in *G*. *m*. *centralis* range [63]. Low *G*. *m*. *centralis* probabilities of occurrence associated with an increase in precipitation of the driest quarter could be attributed to a reduction in leaf cover that serves to protect larviposition sites from direct rainfall and causes pupal mortality by drowning.

The correlation between land cover and the geographic distribution of tsetse is well established [10,15,64]. Land cover characteristics influence all major aspects of tsetse ecology such as the provision of suitable microclimatic conditions, the presence of suitable resting and larviposition sites, and the availability of wild and domestic hosts [10]. For *G*. *m*. *morsitans*, high probabilities of occurrence were associated with riverine woody savannah that is known to be the dry season home of this subspecies [65]. Riverine woodland maintains suitable humidity conditions that prevent adult desiccation at high temperatures. High *G*. *m*. *centralis* probabilities of occurrence were associated with deciduous Miombo woodland which buffers microclimates within its range by maintaining the mean temperature of the coldest month at 16.9°C and mean temperature of the hottest month at 23.3°C [63]. These temperatures are conducive to both pupal development and adult survival [59].

Our results further indicated that habitat suitability of *G*. *m*. *morsitans* and *G*. *m centralis* are determined by different environmental variables in agreement with the finding of Robinson et al. [66] and Rogers and Robinson [67]. This suggests that these allopatric subspecies have adapted to different environmental conditions that occur within their specific geographic ranges. Whether this adaptation is due to phenotypic plasticity (that ability of a genotype to produce different phenotypes when exposed to different environmental conditions [68] or has a genetic basis is yet unclear [69]. The implication for ecological niche modelling of *G*. *morsitans* is that its subspecies should be modelled separately whether predicting current distributions or projecting in space and time.

The predicted potential distribution of *G*. *m*. *centralis* and *G*. *m*. *morsitans* under current environmental conditions was observed to have reduced by 47 and 29%, respectively. The reduction could be attributed to an increase in temperature and land cover change in areas that were previously deemed suitable. Lord et al [60] showed that temperature increases of around 2°C between 1995 and 2017 could explain an estimated 90% reduction in tsetse abundance in the Zambezi Valley of Zimbabwe. Temperature-related climate change was also implicated in the spatial changes in tsetse distributions within this area [70]. Similar temperature changes may be responsible for the observed reduction in *G*. *morsitans* distribution in this study as temperatures throughout the African continent have risen by 1.5°C since 1900 [71]. Land cover changes dues to the gradual clearing of natural vegetation for cultivation, the introduction of domestic animals, and the expansion of human settlements have led to a substantial reduction in and fragmentation of natural tsetse habitat [72]. In Zambia, increased tsetse habitat fragmentation has been associated with a reduction in *G*. *morsitans* apparent density [12]. Habitat fragmentation reduces the ability of vegetation to buffer microclimates from climatic variation outside the tsetse habitat [73] and results in climatic conditions that reduce the abundance of flies. Deforestation, on the other hand, results in the loss of suitable resting and larviposition sites [74] and causes changes in climatic variables important for tsetse development [73].

We conclude that the spatial distribution of *G*. *morsitans* in Zambia has reduced by an estimated 101,051 km^2^. Tsetse densities are expected to be high where natural vegetation and wildlife are protected from anthropogenic influence, and suitable climatic conditions exist. Such areas are primarily located within and around national parks and game management areas. Biting intensity is therefore expected to be highest in these areas with increased risk of HAT transmission to tourists, wildlife scouts, and other individuals who utilize these areas for their economic survival. Further, AAT transmission is likely to be highest in cattle populations that graze at the interphase between open and protected lands. We, therefore, recommend that vector management strategies should be adjusted to account for the observed change in *G morsitans* distribution. We further conclude that VST is effective for sampling *G*. *morsitans* outside experimental settings and recommend its’ incorporation as an additional tsetse survey tool.

## Supporting information

S1 Fig *G*. *m*. *morsitans* predictor correlation plot. (TIF)

S2 Fig *G*. *m*. *centralis* predictor correlation plot. (TIF)

S3 Fig Department of National Parks and Wildlife Zambia Permit. (PDF)

S4 Fig *G*. *m*. *morsitans* raw survey results. (CSV)

S5 Fig *G*. *m*. *centralis* raw survey results. (CSV)
