# Supplementary figures and images for: Spatial distribution of Glossina morsitans (Diptera: Glossinidae) in Zambia: A vehicle-mounted sticky trap survey and Maxent species distribution model
Source: PLoS Negl Trop Dis. 2023 Jul 27;17(7):e0011512. doi: 10.1371/journal.pntd.0011512 (PMC10409263; doi:10.1371/journal.pntd.0011512)

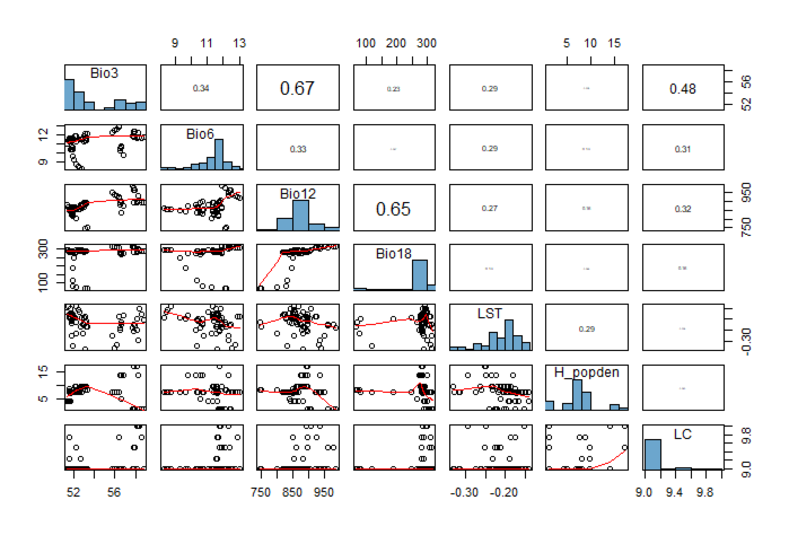

Supplement: S1 Fig — (TIF) [file pntd.0011512.s001.tif]

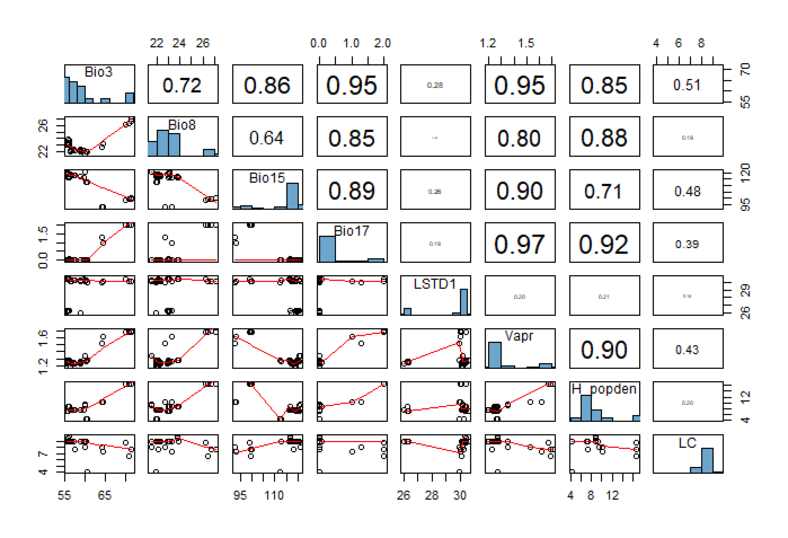

Supplement: S2 Fig — (TIF) [file pntd.0011512.s002.tif]
