# Supplementary material for: Spatial distribution of Glossina morsitans (Diptera: Glossinidae) in Zambia: A vehicle-mounted sticky trap survey and Maxent species distribution model
Source: PLoS Negl Trop Dis. 2023 Jul 27;17(7):e0011512. doi: 10.1371/journal.pntd.0011512 (PMC10409263; doi:10.1371/journal.pntd.0011512)
Supplement: S3 Fig — (PDF) [file pntd.0011512.s003.pdf]

NPW/3/25/1

15th July 2021

REPUBLIC OF ZAMBIA
MINISTRY OF TOURISM AND ARTS
DEPT OF NATIONAL PARKS AND WILDLIFE
7 NOV 2021
WEST LUNGA AREA MANAGEMENT UNIT
AREA WARDEN'S OFFICE
P.O BOX 130077, MUFUMBWE.

The Chief Tsetse Control Biologist
Ministry of Fisheries and Livestock
Department of Veterinary Services
Tsetse and Trypanosomiasis Control Unit
P.O Box 350001
CHILANGA

RE: REQUEST FOR PERMISSION TO SAMPLE TSETSE FLIES IN AND AROUND NATIONAL PARKS

Reference is made to your letter dated 12th July 2021 concerning the above subject.

I am pleased to inform you that you have been granted permission to sample tsetse flies in and around all National Parks in the country. Sampling of tsetse flies will be based on a vehicle-mounted sticky trap, which will be driven along motorable tracks in and around national parks. This sampling activity will enable the mapping of the spatial distribution of tsetse flies, which are important vectors of African trypanosomiasis. The permit is valid from 19th July 2021 to 30th November 2021.

This permit is granted to the following people:

NAME	ID	NATIONALITY
Emmanuel Banda	932103/11/1	Zambian
Milner Mukumbwali	297970/74/1	Zambian
Jackson Muyobela	797129/11/1	Zambian

Cleared
Francis Samalum[illegible]
AREA WARDEN
07/11/2021

The permit is granted on the following conditions:

1. You shall conduct the assessment under the supervision of the Veterinarian and Ecologists in the respective national parks at your own cost.
2. You shall adhere to all rules and regulations when in the National Parks and Game Management Area.
3. You shall submit a copy of the research results and report to DNPW before publication and ensure that all publications from this work are co-authored with DNPW.

4. You shall be escorted into the park by a Wildlife Police Officer at all times and at your own cost.
5. No Unmanned Aerial Vehicles shall be used in this study.
6. The permit is subject to any other written laws of Zambia.

Kindly, note that you may be requested to make an oral presentation to DNPW of your research findings and its conservation and management implications.

Yours sincerely,

Chuma Simukonda, DSc
DIRECTOR - NATIONAL PARKS AND WILDLIFE

Cc: Acting Assistant Director - Research and Veterinary Services
Cc: Senior Wildlife Wardens – All Regions
